# Cycloastragenol promotes dorsal column axon regeneration in mice

**DOI:** 10.3389/fncel.2024.1424137

**Published:** 2025-01-03

**Authors:** Tao Zihan, Teng Wenwen, Ma Yanxia, Saijilafu

**Affiliations:** ^1^Key Laboratory of Novel Targets and Drug Study for Neural Repair of Zhejiang Province, School of Medicine, Hangzhou City University, Hangzhou, China; ^2^Orthopaedic Institute, Suzhou Medical College, Soochow University, Suzhou, Jiangsu, China

**Keywords:** cycloastragenol, dorsal column, axon growth, nerve injury, functional recovery, TERT, spinal cord injury, p53

## Abstract

**Introduction:**

Cycloastragenol (CAG) has a wide range of pharmacological effects, including anti-inflammatory, antiaging, antioxidative, and antitumorigenic properties. In addition, our previous study showed that CAG administration can promote axonal regeneration in peripheral neurons. However, whether CAG can activate axon regeneration central nervous system (CNS) remains unknown.

**Methods:**

Here, we established a novel mouse model for visualizing spinal cord dorsal column axon regeneration involving the injection of AAV2/9-Cre into the lumbar 4/5 dorsal root ganglion (DRG) of Rosa-tdTomato reporter mice. We then treated mice by intraperitoneal administration of CAG.

**Results:**

Our results showed that intraperitoneal CAG injections significantly promoted the growth of *vitro*-cultured DRG axons as well as the growth of dorsal column axons over the injury site in spinal cord injury (SCI) mice. Our results further indicate that CAG administration can promote the recovery of sensory and urinary function in SCI mice.

**Conclusion:**

Together, our findings highlight the therapeutic potential of CAG in spinal cord injury repair.

## Introduction

Cycloastragenol (CAG) is the active form of astragaloside IV isolated from *Astragalus membranaceus* (Fu et al., 2014; Li M. et al., 2020; Lin et al., 2022). This triterpenoid has a wide range of pharmacological effects, including anti-inflammatory (Bagalagel et al., 2022; Melin et al., 2022; Zhao et al., 2015; Zhu et al., 2021), antiaging (Cheng et al., 2020; Zhang et al., 2023), antioxidative (Yu et al., 2018), and antitumorigenic (Deng et al., 2022; Yang et al., 2022) properties. For example, CAG reduces tumor necrosis factor-alpha (TNF-α) levels by inhibiting TXNIP/NLRP3 inflammasome activation, thus exerting antioxidative activities (Zhao et al., 2015). Additionally, recent studies on colorectal cancer organoids have revealed that CAG enhances the tumor-killing ability of CD8 + T cells by binding to cathepsin B and thereby inhibiting the degradation of major histocompatibility complex I (MHC-I) (Deng et al., 2022; Yang et al., 2022). Meanwhile, regarding its neurological effects, CAG was shown to upregulate the expression of nerve growth factor (NGF) in the cerebral cortex of mice (Ip et al., 2014) as well as, promote the proliferation and survival of neural stem cells *in vitro* (Yu et al., 2018).

The World Health Organization (WHO) has estimated that 250,000 to 500,000 people worldwide suffer a spinal cord injury (SCI) annually, with up to 90% of these cases being due to trauma. SCI often leads to the loss of motor and sensory functions, and restoring nerve function after SCI remains challenging. However, there are currently no effective treatments for SCI. In the central nervous system (CNS) of adults, neurons have limited regenerative ability, and damage often triggers an inflammatory response that further hinders axon regeneration (Bodnar et al., 2021; Kotaka et al., 2017; Singh, 2022). In addition, when the spinal cord is damaged, cells in the affected region release reactive oxygen species (ROS), leading to the recruitment of a series of proinflammatory cytokines, such as TNF-α, which can exacerbate mitochondrial damage and DNA oxidation, forming a vicious cycle that eventually leads to spinal cord cell apoptosis (Liu D. et al., 2023; Liu et al., 2020; Yin et al., 2024). These proinflammatory factors contribute to the formation of glial scars, which act as a physical barrier to axon regeneration in the spinal cord (Li Y. et al., 2020; Liu et al., 2021).

Given that it has been demonstrated that CAG can inhibit ROS production (Yu et al., 2018), in this study, we investigated whether CAG can promote SCI repair *via* its antioxidative activities. For this, we established a novel mouse model for visualizing spinal cord dorsal column regeneration involving the injection of AAV2/9-Cre into the lumbar 4/5 dorsal root ganglion (DRG) of Rosa-tdTomato reporter mice. Once Cre recombinase is expressed, the LoxP site in the Rosa-tdTomato mouse genome will be recognized and excised, resulting in the expression of tdTomato, a red fluorescent protein. This enables the visualization of whole axons as well as the tracking of the growth of individual axons within the sciatic nerve and spinal cord dorsal column. Previously, we showed that CAG administration can promote axonal regeneration in peripheral neurons *in vitro* (Ma et al., 2019). However, whether CAG can activate axon regeneration *in vivo* remains unknown. Here, we found that intraperitoneal CAG injections significantly promoted the growth of *vitro*-cultured DRG axons as well as the growth of dorsal column axons over the injury site in SCI model mice. Our results further indicate that CAG administration can promote the recovery of sensory, and urinary function in SCI mice. Together, our findings highlight the therapeutic potential of CAG in spinal cord injury repair.

## Materials and methods

### Animals

Adult ICR and Rosa-tdTomato^*f*/*f*^ reporter mice were used in this study. All experiments were performed on female mice aged between 6 and 8 weeks. All the animals were housed in a specific pathogen-free (SPF)-rated animal facility with a room temperature of between 20 and 26°C and a relative humidity of 40% to 70%. Each cage contained five animals, and the mice were provided with an adequate diet and clean drinking water. All protocols involving animals were approved by the Animal Experiment Ethics Committee of Soochow University (Approval No.: SUDA20240307A03). Surgery was performed under deep anesthesia and every effort was made to minimize the suffering of the animals.

### Drug administration

Mice were assigned to two groups—a Control group, in which the animals were intraperitoneally injected with a solution containing phosphate-buffered saline (PBS), 2% Tween 20, and 5% DMSO; and an experimental group, in which the mice were intraperitoneally injected with the same solution supplemented with CAG at a concentration of 2.5 mg/mL. The mice were dosed twice daily, with a single dose set at 20 mg/kg. The CAG used in this study (S26736, Yuanye Bio-Technology, Shanghai, China) had a purity of ≥98% and the solvents used for preparation were of sterile grade.

### Cell culture

The L4–L6 DRG was removed and digested first with collagenase for 1.5 h and then with TrypLE (Gibco, 25200-056, USA) for 15 min. Then, washed with Minimum Essential Medium (MEM) supplemented with 10% fetal bovine serum (FBS) to terminate the digestion, and dissociated with a 1-mL pipette. Subsequently, the cells were cultured in 24-well plates for 48 h at 37°C with 5% CO_2_. The cell culture medium consisted of basal medium (Gibco, 21103049), penicillin/streptomycin, 1 × GlutaMAX (Gibco, 35050061), and B-27 supplement (Gibco, 17504044). Cell morphology was analyzed using a Zeiss microscope (Carl Zeiss, AXIO, Germany) and axon length was measured using AxioVision 4.7 software. To assess the cell survival rate, the average number of Tuj1-positive cells per square millimeter was calculated using Image J software.

### Dorsal column crush

Following anesthesia with 100 mg/kg ketamine and 10 mg/kg xylazine, the spinal cord and the L4 DRG of the mice were exposed *via* laminectomy. Under a microscope, 1 μL of AAV2/9-Cre (HANBIO, 71090612, China) was injected into the DRG using the Picospritzer III instrument (Parker Hannifin, Cleveland, OH, USA) and a glass needle. Then, at the level of T12, the spinal cord was crushed for 1s using custom-modified 5^#^ forceps, after which the muscles and skin were sutured with 4-0 nylon sutures. The bladder of each animal was squeezed every day to ensure daily urination (The number of animals subjected to each procedure is six, *n* = 6).

### Immunofluorescence staining

Cells were fixed in 4% paraformaldehyde (PFA) for 20 min at room temperature and blocked with 2% BSA for 60 min. For tissue section staining, animals were perfused with 4% PFA, and the samples were dissected out. Tissue samples were cryosectioned at a thickness of 12-μm and blocked with 10% FBS and 0.3% Triton X-100 for 1 h. Then, the samples (cells and tissue) were incubated at room temperature for 90 min with primary antibodies and with secondary antibodies for 60 min, also at room temperature. The primary antibodies used were GFAP Rabbit mAb (1:200, CST, 80788, USA), Tuj1 Rabbit polyclonal (1:200, Sigma, T2200, USA), Tuj1 Mouse mAb (1:1000, Biolegend, 801202, USA), TGF-beta Rabbit Ab (1:200, CST, 3711S), TNF-alpha Rabbit mAb (1:200, CST, 11948S), iNOS Rabbit mAb (1:200, CST, 13120S). The secondary antibodies included Alexa 488 goat anti-rabbit IgG (Invitrogen, A11008, USA), Alexa 488 goat anti-mouse IgG (Invitrogen, A11001), Alexa 594 goat anti-mouse IgG (Invitrogen, A11005), and Alexa 594 goat anti-rabbit IgG (Invitrogen, A11012).

Cryosection and cultured cell images were acquired using an inverted light microscope (Zeiss Axiovert 200, Carl Zeiss MicroImaging), which was outfitted with epifluorescence optics and a charge-coupled device (CCD) camera. The microscope was operated using Axiovision software (Carl Zeiss MicroImaging) to control the CCD camera and capture the images.

### Western blot

Samples were lysed with RIPA buffer (Beyotime, China) and total protein concentrations were determined with a BCA kit (Beyotime). Proteins were separated using sodium dodecyl sulfate–polyacrylamide gel electrophoresis, transferred to a polyvinylidene fluoride membrane, blocked with 5% skimmed milk powder, and incubated first with primary antibodies for 12 h at 4°C (GAPDH Rabbit mAb [1:1000, CST, 2188], β-actin Rabbit mAb [1:1000, CST, 4970], TERT Rabbit mAb [1:200, Abcam, ab32020], p53 Mouse mAb [1:200, Abcam, ab26]) and then with secondary antibodies at room temperature for 1 h. Bands were developed with Immobilon Western Chemiluminescent HRP Substrate (MILLIPORE, 638173, USA) and band intensity was quantified using ImageJ software.

### Behavioral tests

Hot plate experiments: The pain threshold for thermal stimulation in mice was determined using an X2026TBD intelligent thermostatic hot plate instrument. The hot plate was preheated to 55 ± 0.1°C. Mice were placed on the surface of the hot plate and the time it took for them to start jumping or licking the hindfoot was measured and used as a pain threshold indicator. To prevent foot burns, mice were removed from the hot plate after 40 s; the pain threshold was calculated as 40 s for mice that did not jump or lick their hind feet during this time.

### Micro-CT scanning

Mice were scanned with the NEMO Micro-CT (Pingsheng scientific, Kunshan, China) at the same time every day. The parameters were set as follows: Tube voltage 60 kV, tube current 0.11 mA, imaging speed 20 frames/s, transverse field-of-view (fov) 50 mm, and axial fov16 mm. After data reconstruction, the maximum cross-section of the bladder was selected for comparative analysis.

### Quantitative real-time PCR

For total RNA extraction, samples were homogenized in TRIzol Reagent (Invitrogen,15596026) and precipitated with chloroform, isopropanol, and 75% ethanol. The RNA concentration was measured using a NanoDrop at 4°C. The mRNA was reverse transcribed into cDNA (42°C for 60 min, 70°C for 5 min) using the RevertAid First Strand cDNA Synthesis Kit (Thermo Scientific, 01016280, USA). Quantitative real-time PCR was performed with iTaq Universal SYBR Green Supermix (1725124, USA) using the following cycling conditions: 95°C for 10 s for initial denaturation, followed by 40 cycles of denaturation at 95°C for 15 s, annealing at 55°C for 30 s, and extension at 72°C for 30 s. The fold change of the genes of interest were calculated using the 2^–ΔΔCt^ method. The primer sequences were as follows (5′–3′):

*Tp53*-F: CGA CGA CAT TCG GAT AAG, *Tp53*-R: TTG CCA GAT GAG GGA CTA;
*Tert*-F: TGG TGG AGG TTG CCA, *Tert*-R: CCA CTG CAT ACT GGC GGA TAC;
*Tnf*-F: ATG TCT CAG CCT CTT CTC ATT C, *Tnf*-R: GCT TGT CAC TCG AAT TTT GAG A;
*Il10*-F: TTC TTT CAA ACA AAG GAC CAG C, *Il10*-R: GCA ACC CAA GTA ACC CTT AAA G;
*Tgfb*-F: CCA GAT CCT GTC CAA ACT AAG G, *Tgfb*-R: CTC TTT AGC ATA GTA GTC CGC T;
*Gapdh*-F: AGG AAT TGA CGG AAG GGC ACC, *Gapdh*-R: GTG CAG CCC CGG ACA TCT AAG;
*Il1b*-F: CAC TAC AGG CTC CGA GAT GAA CAA C, *Il1b*-R: TGT CGT TGC TTG GTT CTC CTT GTA C.

### ROS assay

After washing with PBS, the samples were incubated with 10 μM dihydroethidium (Beyotime) at 37°C for 1 h, incubated with DAPI for 15 min, washed with PBS, and mounted with Mowiol.

### Statistical analysis

All data are presented as means ± standard deviation (SD) and were analyzed using GraphPad Prism 8.0.2 software. Comparisons among three or more groups were performed by one-way ANOVA. The student’s *t*-test was used for comparisons between two groups. A *p*-value < 0.05 was considered significant.

## Results

### CAG promoted axon regeneration in peripheral sensory neurons

**FIGURE 1:**
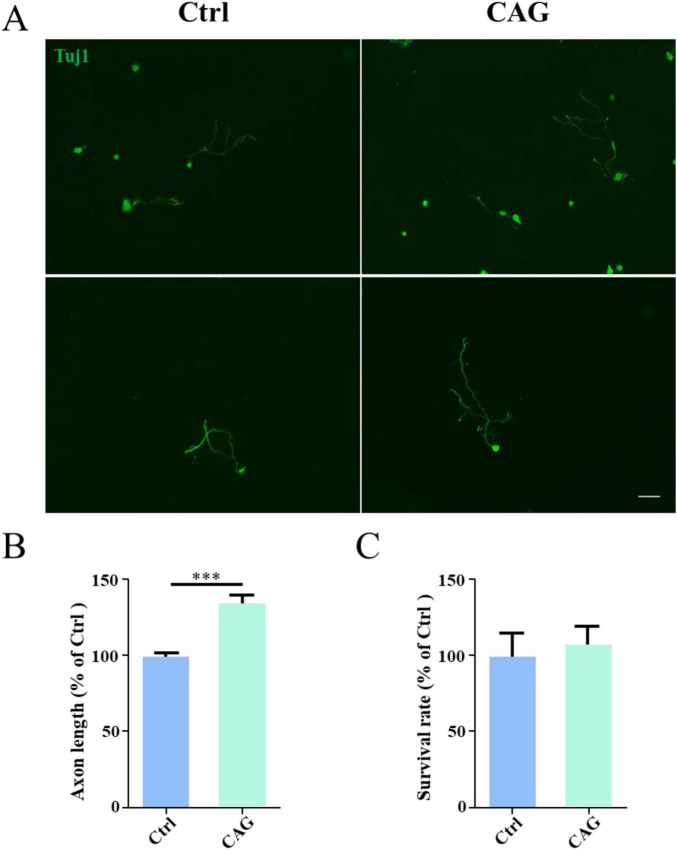
Cycloastragenol (CAG) promotes axon regeneration in peripheral sensory neurons. **(A)** Representative images of Tuj1 (green) immunofluorescence staining in dorsal root ganglion (DRG) neurons obtained from mice after 7 days of CAG administration and subsequently cultured for 48 h. Scale bar = 100 μm. **(B)** Axon length measurements showed that the *in vivo* administration of CAG promoted axon growth in *in vitro*-cultured DRG neurons (*n* = 3, ***p < 0.001). **(C)** CAG administration did not affect the survival of DRG neurons after 3 days of culture (*n* = 3).

We first investigated the promotive effect of CAG on peripheral nerve axon growth. For this, CAG was administered to adult ICR mice (twice daily at a single dose of 20 mg/kg) by intraperitoneal injection. After 7 days, DRG sensory neurons were obtained from mice, and cultured *in vitro* for 48 h. As shown in Figures 1A, B, the administration of CAG accelerated the growth of DRG axons without affecting neuronal survival (Figure 1C). These results indicated that CAG could promote axonal growth in peripheral sensory neurons.

### A novel model for visualizing dorsal column regeneration

**FIGURE 2:**
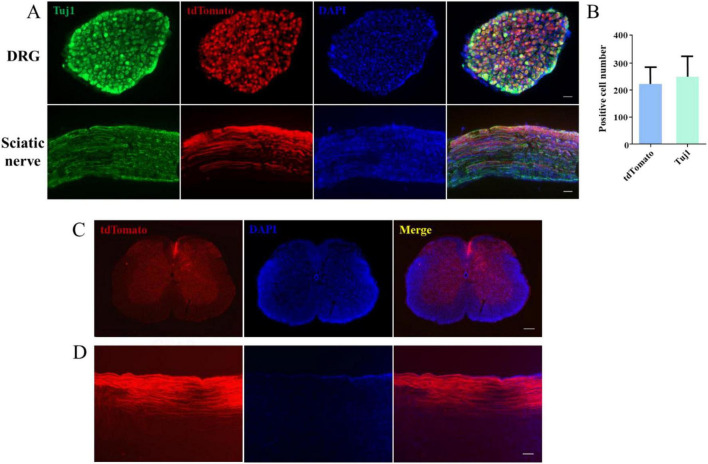
A novel model for the visualization of dorsal column axon regeneration. **(A)** Immunofluorescence staining of dorsal root ganglion (DRG) and sciatic nerve sections showed a high DRG sensory neuron transfection rate 4 weeks after the injection of AAV2/9-Cre into the lumbar (L) 4/5 DRG of ROSA-tdTomato floxed mice. Green: Tuj1 staining, red: transfected sensory neurons, blue: DAPI staining. Scale bar = 100 μm (*n* = 3). **(B)** Quantification of the transfection rate in panel **(A)**. **(C)** Immunofluorescence staining of a spinal cord cross-section showing the presence of numerous tdTomato-labeled axons in the dorsal column 6 weeks after AAV2/9-Cre injection into the L4–5 DRG of ROSA-tdTomato floxed mice. Red: tdTomato-positive axons, blue: DAPI staining. Scale bar = 200 μm. **(D)** Immunofluorescence staining of a sagittal section of the spinal cord showing the presence of numerous tdTomato-positive axons in the dorsal column 6 weeks after AAV2/9-Cre injection into the L4–5 DRG of ROSA-tdTomato floxed mice; red: tdTomato-positive axons, blue: DAPI staining. Scale bar = 35 μm.

Next, to investigate whether CAG can stimulate SCI repair, we established a novel model for visualizing dorsal column axon regeneration using the Cre recombinase system. AAV2/9-Cre was injected into the lumbar 4/5 DRG of Rosa-tdTomato floxed mice, allowing tdTomato protein to be expressed in DRG neurons following Cre-mediated recombination. We traced tdTomato-positive axons in the peripheral nervous system (PNS) and found that approximately 84% of DRG neurons were fluorescently labeled (Figures 2A, B). Individual tdTomato-positive axons were also detected in the sciatic nerve (Figure 2A). Additionally, we observed numerous tdTomato-labeled axons in both cross-sections (Figure 2C) and sagittal sections (Figure 2D) of the dorsal column.

### CAG promoted axon regeneration in the dorsal column after SCI

**FIGURE 3:**
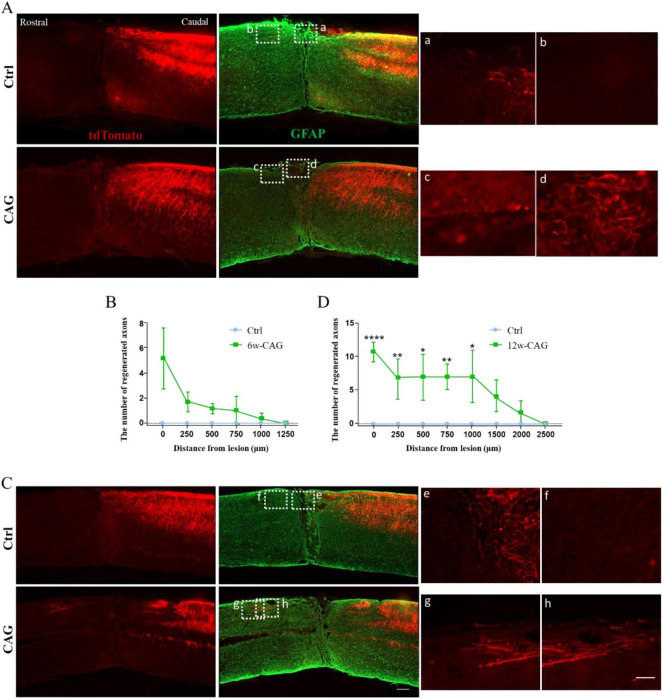
Cycloastragenol (CAG) promotes axon regeneration in the dorsal column of mice after spinal cord injury (SCI). **(A)** After 6 weeks of intraperitoneal CAG injection, immunofluorescence staining of sagittal sections of the spinal cord showed that relatively few tdTomato-positive axons had grown over the lesion site in the dorsal column. All the tdTomato-positive axons stopped in the caudal part of the injury site in both control and CAG-treated mice. Green: GFAP staining. Scale bar = 100 μm. **(B)** Statistics for the axon length across the lesion site in panel **(A)** (*n* = 6). **(C)** After 12 weeks of intraperitoneal CAG injection, immunofluorescence staining of sagittal sections of the spinal cord showed that a greater number of tdTomato-positive axons had grown over the lesion site in the dorsal column and had entered the rostral part of the lesioned spinal cord compared with that seen in the control group. Green: GFAP staining. Scale bar = 100 μm. **(D)** Statistics for the axon length across the lesion site in panel **(C)** (*n* = 6, *p < 0.05, **p < 0.01, ****p < 0.0001).

Although we have previously shown that CAG can promote axon regeneration in peripheral neurons (Ma et al., 2019), whether it can exert similar effects in the CNS remains unknown. Thus, we next investigated whether CAG could promote axonal growth in the dorsal column of the spinal cord using our newly established model. After 6 weeks of intraperitoneal CAG injection, almost no tdTomato-positive axons were detected in the rostral part of the lesion site either in control or CAG-treated mice (Figures 3A, B). However, after 12 weeks of treatment, some tdTomato-positive axons were observed over the lesion site, with the longest tdTomato-positive axons extending 1,500 μm rostral to the crush site (Figures 3C, D). These results indicated that CAG promotes axon regeneration in the dorsal column *in vivo*.

### CAG promoted the recovery of sensory function in SCI model mice

**FIGURE 4:**
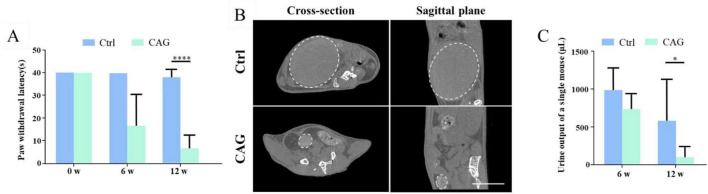
Cycloastragenol (CAG) promotes the recovery of both sensory functions in spinal cord injury (SCI) mice. **(A)** The results of the hot plate experiment showed that CAG administration markedly promoted the recovery of sensory function in the hind limbs of mice 6 and 12 weeks after SCI (*n* = 6, *p < 0.05, ***p < 0.001, ****p < 0.0001). **(B)** Representative micro-CT image showing that, compared with control mice, bladder size was significantly reduced in CAG-treated mice after 12 weeks of treatment (*n* = 5), Scale bar = 10 mm. **(C)** Quantification results showed that the urine retention volume was significantly decreased in SCI mice after 6 and 12 weeks of CAG administration, (*n* = 6, *p < 0.05).

Next, we investigated the effect of CAG on the recovery of sensory function in mice using the hot plate experiment. After 6 weeks of CAG administration, there was no difference in the recovery of sensory nerve function between control and CAG-treated mice (Figure 4A). However, after 12 weeks of treatment, sensory nerve function was significantly better in CAG-treated mice than in control animals (Figure 4A), while the urine retention volume was significantly lower in the CAG group than in the control group (Figures 4B, C). Consistent with these observations, CT scans also showed that bladder size in the CAG group was significantly reduced compared with that in the control group after 12 weeks of CAG treatment (Figures 4B, C). This data indicated that CAG could promote the recovery of sensory function and urinary system function.

### CAG upregulated the expression of telomerase reverse transcriptase (TERT) and p53

**FIGURE 5:**
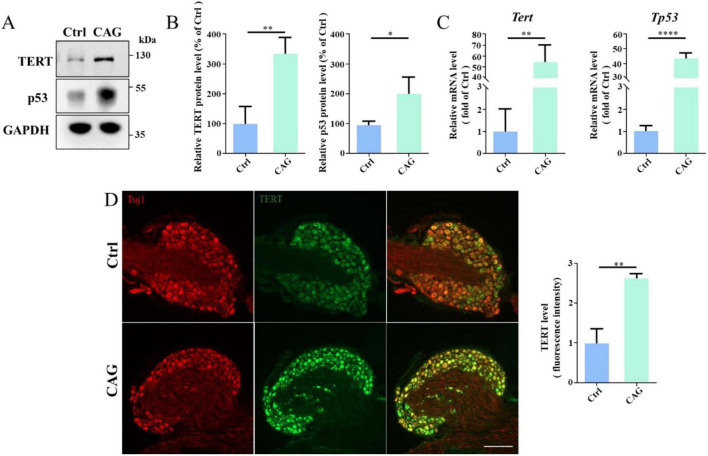
Cycloastragenol (CAG) upregulates TERT and p53 expression. **(A)** Representative western blot showing that TERT and p53 expression levels were increased in sensory neurons after 7 days of intraperitoneal CAG injection. **(B)** Quantification of TERT and p53 protein contents in panel **(A)**, (*n* = 3, *p < 0.05, **p < 0.01). **(C)** Quantitative PCR analysis showed that TERT and p53 mRNA levels in sensory neurons were increased after 7 days of intraperitoneal CAG injection, (*n* = 3, **p < 0.01, ****p < 0.0001). **(D)** Immunofluorescence staining showed that TERT expression was increased in dorsal root ganglion (DRG) sensory neurons after 7 days of intraperitoneal CAG injection. Scale bar = 100 μm (*n* = 3, **p < 0.01).

It has been reported that CAG can upregulate TERT expression in the PNS (Ip et al., 2014; Ma et al., 2019), and TERT and p53 could regulates the PNS axon regeneration (Ma et al., 2019). Accordingly, we determined whether CAG could also induce TERT and p53 expression in the dorsal column axon regeneration. Western blot analysis showed that, after 7 days of CAG administration, TERT and p53 protein levels in DRG were increased in the CAG group compared with that in the controls (Figures 5A, B). In line with these findings, the mRNA levels of *Tert* and *Tp53* in DRG were also significantly increased after 7 days of CAG treatment (Figure 5C). Similarly, immunofluorescence staining also supported that CAG upregulates TERT expression in DRG (Figure 5D). Combined, these findings suggested that CAG may mediate dorsal column axon regeneration by upregulating TERT and p53 expression in the soma of DRG neurons.

### CAG reduced the inflammatory response at the site of SCI

**FIGURE 6:**
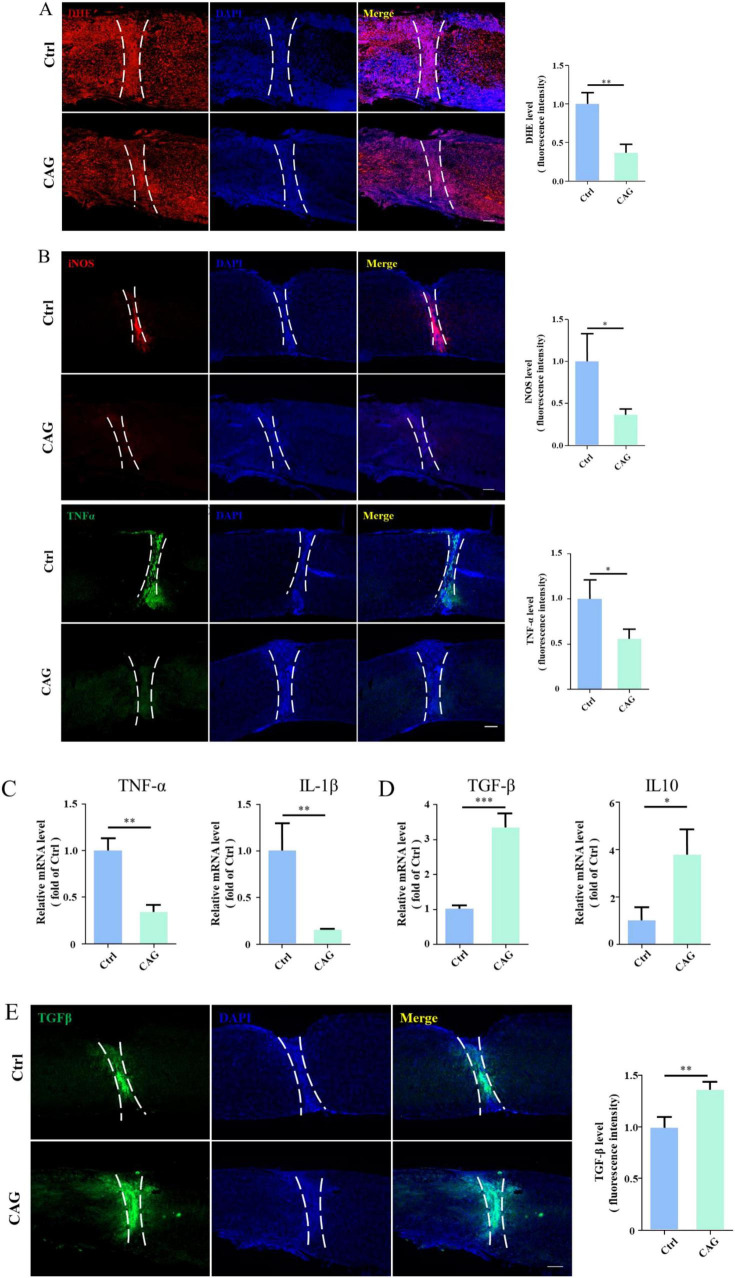
Cycloastragenol (CAG) suppressed the inflammatory response at the site of spinal cord injury (SCI). **(A)** Immunofluorescence staining showed that reactive oxygen species (ROS) contents at the site of SCI were decreased after 7 days of intraperitoneal CAG injection, (*n* = 3, **p < 0.01). **(B)** Immunofluorescence staining showed that TNF-α and iNOS expressions at the site of injury was decreased after 7 days of intraperitoneal CAG administration, (*n* = 3, *p < 0.05). **(C)** The mRNA expression of *Tnf-*α and *Il-1*β was decreased at the site of injury after 7 days of intraperitoneal CAG injection, (*n* = 3, **p < 0.01). **(D)** The mRNA expression of *Tgf-*β and *Il10* at the site of SCI was elevated after 7 days of intraperitoneal CAG injection, (*n* = 3, *p < 0.05, **p < 0.001, ***p < 0.001). **(E)** Immunofluorescence staining showed that TGF-β expression at the site of injury was elevated after 7 days of intraperitoneal CAG administration, (*n* = 3, **p < 0.01).

Local inflammation induced by injury is one of the factors limiting spontaneous axon regeneration after SCI. It has been reported that mitigating oxidative stress or reducing the release of proinflammatory cytokines is beneficial for SCI repair (Yu et al., 2018). Additionally, studies have shown that CAG has anti-inflammatory properties (Zhao et al., 2015). Consequently, we next explored whether CAG administration can reduce the inflammatory response at the site of SCI. We found that the level of dihydroethidium (DHE, a ROS probe) at the SCI site was significantly lower in CAG-treated mice than in control animals after 7 days of treatment (Figure 6A). Furthermore, the expression levels of proinflammatory cytokines, such as TNF-α and inducible nitric oxide synthase (iNOS), were significantly lower in CAG-treated mice than in the controls (Figure 6B), as were the mRNA levels of *Tnf*-α and *Il-1*β (Figure 6C). In contrast, the mRNA levels of the anti-inflammatory cytokines *Il10* and *Tgf-*β were upregulated following CAG administration (Figures 6D, E). These results suggested that CAG inhibits the inflammatory response at the site of SCI.

## Discussion

Axons in the CNS have limited regenerative ability after injury (Saijilafu et al., 2013). DRG contain the cell bodies of primary sensory neurons, which are responsible for transmitting and receiving sensory stimuli, such as touch and pain (Hill and Bautista, 2020; Lin and Chen, 2018; Nguyen et al., 2021). DRG neurons are pseudo-unipolar; their cell body extends a short axon, which then divides into two branches, namely, a peripheral, descending axon branch that innervates peripheral sensory receptors, and an ascending central branch that projects into the dorsal column of the spinal cord to convey sensory information to the brain (Saijilafu et al., 2011). Interestingly, the peripheral branches of DRG neurons can efficiently regenerate after injury. Damage to the peripheral branches of DRG neurons can activate the expression of regeneration-associated genes in their soma, such as *GAP-43*, *ATF3*, and *SMAD1* (Parikh et al., 2011; Seijffers et al., 2007; Van der Zee et al., 1989). However, damage to the central branches of DRG neurons in the dorsal column does not lead to the activation of their intrinsic axon-regenerating ability, thereby limiting their regeneration after injury. In this study, we established a novel experimental model for investigating axon regeneration in the dorsal column of the spinal cord *via* Cre-mediated recombination. The induction of the Tomato protein expression in DGR neurons allowed the visualization of the morphology of the entire cell, including both peripheral and central branches. Additionally, by tracing tdTomato fluorescence, we could clearly measure or detect regenerating central branch axons in the dorsal column. After the injection of AAV2/9-Cre into the lumbar 4/5 DRG of Rosa-tdTomato floxed mice, we found that approximately 84% of DRG neurons expressed tdTomato fluorescent protein. In addition, individual tdTomato-positive axons could be clearly observed in the sciatic nerve, indicating that our experimental model was also useful for studying peripheral sensory axon regeneration. Importantly, our method is more efficient and easier to execute compared with traditional electroporation methods. It was reported that the efficiency of DRG transfection using electroporation is only approximately 43% (Saijilafu et al., 2011). In addition, in rats, the injection of choleragenoid conjugated to horseradish peroxidase (B-HRP) into the sciatic nerve has also been used to retrogradely label regenerating axons in the dorsal column (Neumann and Woolf, 1999). However, the associated dye leakage often reduces the labeling rate, and sometimes further staining is required to visualize dye-positive axons. Therefore, our new method not only simplifies the experimental procedure but also greatly improves its accuracy and reproducibility.

CAG is a triterpene saponin that is increasingly associated with a wide range of pharmacological activities, including the regulation of oxidative stress, neuroinflammation, and apoptosis (Ikram et al., 2021; Yang et al., 2023). CAG can reportedly stimulate the transcriptional activity of farnesoid X receptor (FXR), a potential drug target for the treatment of non-alcoholic fatty liver disease (Chiang and Ferrell, 2020; Gu et al., 2017). Moreover, CAG can inhibit the DNA-binding activity of STAT3 and prevent its activation in human gastric tumor cells (Hwang et al., 2019). CAG can also upregulate SIRT1 expression as well as inhibit NF-κB activation-induced neuroinflammation (Li M. et al., 2020), indicating that CAG possesses anti-inflammatory properties. SCI often leads to the production of inflammatory factors at the site of injury, which impairs axon regeneration. Here, we also found that CAG reduces ROS generation and blocks the release of proinflammatory cytokines such as TNF-α, IL-1β, and iNOS, which is accompanied by an increase in the expression of the anti-inflammatory cytokines IL-10 and TGF-β. Together, these findings demonstrate that CAG reduces the inflammatory response at the site of SCI, and may accelerate the repair process. Our *in vitro* cell culture data showed that CAG did not affect the survival of DRG neurons. It has been reported that the highest CAG dose administered to rats, 150 mg/kg bodyweight/day, equivalent to 10500 mg/day in a 70-kg human, was non-toxic to the animals (Szabo, 2014). This means CAG were relatively safe for *in vivo* administration.

Several studies have shown that CAG can activate telomerase (Idrees et al., 2023; Ip et al., 2014; Khan et al., 2023; Wang et al., 2022), the enzyme responsible for the maintenance of telomere length in cells (Bernardes de Jesus and Blasco, 2013; Mizukoshi and Kaneko, 2019). Telomerase consists of two subunits, namely, TERT and TERC (telomeric RNA) (Hoffmann et al., 2021; Roake and Artandi, 2020; Wang et al., 2019). CAG can promote HEKn cell survival by inducing TERT expression, thereby activating telomerase (Duman et al., 2021; Ip et al., 2014). CAG can also prevent glucocorticoid-induced osteonecrosis by activating telomerase, rendering it a candidate drug for the treatment of this condition (Wang et al., 2024; Wu et al., 2021). Additionally, TERT regulates the inflammatory response through the NF-κB signaling pathway, and the loss of telomerase activity leads to a significant increase in the production of the inflammatory cytokines TNFα, IL-6, and CCL2 (Deacon and Knox, 2018; Indran et al., 2011; Liu S. et al., 2023; Spilsbury et al., 2015; Wu et al., 2020). In agreement with these reports, we also found that CAG treatment upregulates TERT expression in mouse DRG. TERT is a key regulator of telomerase activity. We and others have previously demonstrated that the inhibition of TERT activity blocks peripheral sensory axon regeneration *in vitro* and *in vivo* (Stenudd et al., 2015; Ma et al., 2019). Thus, it is feasible that CAG promotes dorsal column axon regeneration by activating TERT in the soma of DRG neurons. Using our model, we found that CAG can promote axon regeneration in the injured dorsal column in mice. After 12 weeks of CAG administration, tdTomato-positive axons were clearly seen growing over the lesion site and entering the rostral part of the spinal cord. Behavioral assessments also demonstrated that CAG significantly enhanced the recovery of sensory function.

Despite the importance of our findings, our study had some limitations. Although CAG plays a positive role in SCI repair; however, the underlying detailed molecular mechanism and its functions on motor neuron remains unclear. The regenerative ability of corticospinal tract axons is lower than that of dorsal column axons. Indeed, corticospinal tract neurons have been reported to have the weakest intrinsic axon regeneration ability of all the nerve tracts in the spinal cord (Liu et al., 2010). Additionally, clinical trials should be undertaken to confirm the applicability of CAG in SCI treatment. In summary, our findings showed that CAG has the potential to promote the recovery of patients following SCI, thereby improving their quality of life.

## Institutional review board statement

All experimental procedures and protocols were approved by the Animal Ethics Committee at Soochow University, China (approval No. SUDA20240307A03). All experimental procedures described here were in accordance with the National Institutes of Health (NIH) Guidelines for the Care and Use of Laboratory Animals (NIH Publication No. 85-23, revised 1996).

## Data Availability

The original contributions presented in this study are included in this article/supplementary material, further inquiries can be directed to the corresponding authors.
